# Editorial: Modeling Individual Differences in Perceptual Decision Making

**DOI:** 10.3389/fpsyg.2016.01602

**Published:** 2016-10-25

**Authors:** Joseph W. Houpt, Cheng-Ta Yang, James T. Townsend

**Affiliations:** ^1^Department of Psychology, Wright State UniversityDayton, OH, USA; ^2^Department of Psychology, National Cheng Kung UniversityTainan, Taiwan; ^3^Psychological and Brain Sciences, Indiana University BloomingtonBloomington, IL, USA

**Keywords:** indidvidual differences, perceptual decision-making, multinomial tree models, systems factorial technology, accumulator model

Researchers have been interested in how human beings accumulate and process information for decision-making since the development of experimental psychology in the late nineteenth century and then its renaissance in cognitive science in the 1960s. Whereas psychometrics and test theory, which also got their start in the nineteenth century have made individual differences the foundation of their fields, the study of cognitive processes has traditionally, and over many decades, assumed that the manner in which information is processed for decision-making is invariant across individuals given a particular experimental context.

The typical approach in cognitive psychology has assumed that individual variation affects perceptual processing parametrically (e.g., rate of information accumulation, response bias), but not structurally (e.g., the order of information processing). For example, when using information in working memory, some individuals may be faster, but it is assumed that all individuals use the information in the same manner. With that assumption, the usual practice of developing models is based on grouped data, rather than the individual data.

However, a growing number of studies have demonstrated systematic individual differences in perceptual decision-making. These individual differences can be reflected in both parametric variation corresponding to characteristics of the participants (e.g., working memory span) and structural differences (i.e., in the same task context, different individuals search across visual-spatial information and phonetic information in sequence while others search in parallel). Hence, we as researchers need more complex modeling tools than traditional linear models with null-hypothesis testing to investigate the influences of task, context, and individual differences as well as the potential for interactions among these factors.

In this special issue, we focused on a particular subset of cognitive models that explicitly allow for both structural and parametric variation across individuals, particularly multinomial processing trees, and systems factorial technology (SFT) applied to perceptual decision-making. The motivation for the focus on perceptual decision-making is threefold. Empirical studies of perception have grown out of a history of making a large number of observations for each individual so as to achieve precise estimates of each individual's performance. This type of data, rather than a small number of observations per individual, is most amenable to achieving precision in individual-level and group-level cognitive modeling. Second, the interaction between the acquisition of perceptual information and the decisions based on that information (to the extent that those processes are distinguishable) offers rich data for scientific exploration.

Finally, there is an increasing interest in the practical application of individual variation in perceptual ability, whether to inform perceptual training and expertise, or to guide personnel decisions. That is, some research trajectories seem to be in the process of synthesis of contemporary cognitive psychology with the above mentioned psychometrics tradition.

The contributions of Fific et al., Chechile et al. and Zhang et al. represent fundamental theoretical advances in individual difference modeling.

Chechile's contribution argues for the viability of multinomial processing trees as a more informative model of perceptual decision-making than the traditional signal detection approach. His contribution includes a hierarchical application of the multinomial processing tree model. As signal detection theory is a fundamental tool in perceptual decision-making research, the potential information gain from a multinomial processing tree model could be significant across the field. Furthermore, the hierarchical modeling approach accommodates group-level analysis of individual differences.

Fific's article gives an overview of SFT, a framework that is applied in many of the articles in this special issue, and contributes new analyses and details on the application of SFT. These new contributions include demonstrations of SFT's advantages for studying individual differences and group-level analyses, a consequence of the fact that the diagnostic SFT statistics are estimated at the individual level rather than from data aggregated across subjects. This allows for the empirical investigation of structural individual differences in perceptual decision-making.

Next, Zhang et al. demonstrate a new approach to fitting a particular type of accumulator model to individual subject data: Diffusion models with flexible, time-varying decision boundaries. This approach can reveal individual differences in accumulating evidence toward a decision bound.

Yang and Wu's contribution includes an example of the dangers of averaging data across individuals: Important patterns of performance at the individual level can be obscured when averaging across participants. In their contribution, they argue persuasively that an empirical phenomenon known as the “category variability effect,” which is important for distinguishing among models of perceptual categorization, may be common but often overlooked due to averaging across participants. By applying individual-level modeling, they found clear evidence for the category variability effect in some, but not all, individuals.

Blunden et al. explore individual differences in the effect of categorization training on perceptual discrimination among faces. They use faces generated by combining four different base faces. By applying multiple quantitative approaches (general recognition theory, multi-dimensional scaling, SFT, and the logical-rules framework) they were able to classify individual participants based on whether they use parallel self-terminating processes and what types of interactions occur between the perceptions of each stimulus dimension. This approach leads to a better understanding of individual perceptual categorization training for faces and demonstrates an improved method for exploring individual differences in perceptual categorization in general.

Yu et al. systematically explore the connection between individual variation in SFT capacity measures in three different redundant-target detection tasks and an operations span task score (a commonly used measure of working memory capacity). They find that only the SFT capacity in an audiovisual detection task was positively correlated to the working memory capacity, suggesting that perceptual processing for audiovisual information and the executive function in working memory share similar cognitive resources. The contribution of this study is to demonstrate the use of individual-level modeling to further the understanding of the theoretical links between different levels of capacity measures.

Like Yu et al., Endres et al. focus on connections between SFT capacity measures and individual differences in working memory using parametric models. They develop a new task to examine the relative effects of loading either visual-spatial items or phonetic items into working memory on visual processing capacity as a function of operation span task scores. Standard analyses of response times and accuracy indicated clear differences between individuals with high working memory span and those with low working memory span. Despite this difference, there was no evidence of a difference across groups in the efficiency with which individuals were able to combine the two sources of information. By applying models to the study of individual differences in working memory, Endres et al. better isolate the behavioral locus of working memory deficiencies, which can in turn be used to better understand the mechanism by which working memory varies across individuals.

Houpt et al. examine variation in visual processing capacity as a function of a different construct, reading ability, and particularly dyslexia diagnoses. Building on earlier work measuring word-superiority-type effects across words, pseudowords, and non-words with SFT, they demonstrate how various subpopulations within those diagnosed with dyslexia might be identified. Even with clear differences between those with dyslexia and the control participants on standard diagnostic measures, some of the participants with dyslexia exhibited word superiority effects that were not distinguishable from control participants while others with dyslexia were clearly different. These data inform the current debate about the heterogeneity of dyslexia and indicate that the model-based measure of word superiority may offer additional diagnostic insights.

Nunez et al. explore the connection between cognitive models and EEG measures of attention. They find that individual differences in task performance are explained by parametric variation in an evidence accumulation model. Furthermore, the parametric differences across individuals, particularly in the evidence accumulation rates, are highly correlated with the EEG measure of attentional control.

Chang and Yang's article examines the connection between cultural differences, particularly individual thinking style, and visual processing capacity. Using both accumulator models and SFT, they find that individuals who have higher “middle-way thinking” scores (roughly, the tendency to consider many alternative perspectives) had higher visual processing capacity as well. These findings provide a reasonable cognitive mechanical account for the behavior of high middle-way thinkers. The contribution of this work is to demonstrate that the application of individual-level modeling to study the culture-sensitive behavior.

In sum, many of the “laws” of human thought and behavior garnered over the past one hundred thirty-seven years since Wilhelm Wundt epochally established his laboratory in Leipzig, are based on grouped means and, given the increasing appearance of individual differences in even elementary perceptual, cognitive, and motor tasks, they will likely come under increased scrutiny. Together, the articles gathered in this special issue, demonstrate both the need for models of individual differences in perceptual decision-making and the strength of applying such models. We believe this imposing body of research offers a significant advance toward having the necessary tools for studying the joint influences of task, context, and individual differences on perception.

## Author contributions

All authors listed, have made substantial, direct and intellectual contribution to the work, and approved it for publication.

## Funding

This work was supported by AFOSR FA9550-13-1-0087 awarded to JH, MOST 102-2628-H-006-001-MY3 awarded to CY, and AFOSR FA9550-12-1-0172, NSF 1331047 and NIH-NIMH MH 057717-07 awarded to JT.

### Conflict of interest statement

The authors declare that the research was conducted in the absence of any commercial or financial relationships that could be construed as a potential conflict of interest.

